# Silver Nanoparticles Selectively Treat Neurofibromatosis Type 1-Associated Plexiform Neurofibroma Cells at Doses That Do Not Affect Patient-Matched Schwann Cells

**DOI:** 10.3390/pharmaceutics16030371

**Published:** 2024-03-07

**Authors:** Bashnona Attiah, Garrett Alewine, Mary-Kate Easter, Robert A. Coover, Cale D. Fahrenholtz

**Affiliations:** Department of Basic Pharmaceutical Sciences, Fred Wilson School of Pharmacy, High Point University, High Point, NC 27268, USA

**Keywords:** neurofibromatosis type 1, plexiform neurofibroma, nanomedicine, pediatric cancer, silver nanoparticles

## Abstract

Neurofibromatosis Type 1 (NF1) is a common neurogenic condition characterized by heterozygous loss of function mutations in the neurofibromin gene. NF1 patients are susceptible to the development of neurofibromas, including plexiform neurofibromas (pNFs), which occurs in about half of all cases. Plexiform neurofibroma are benign peripheral nerve sheath tumors originating from Schwann cells after complete loss of neurofibromin; they can be debilitating and also transform into deadly malignant peripheral nerve sheath tumors (MPNSTs). Here, our data indicates that silver nanoparticles (AgNPs) may be useful in the treatment of pNFs. We assessed the cytotoxicity of AgNPs using pNF cells and Schwann cells derived from the same NF1 patient. We found that AgNPs are selectively cytotoxic to pNF cells relative to isogenic Schwann cells. We then examined the role of neurofibromin expression on AgNP-mediated cytotoxicity; restoration of neurofibromin expression in pNF cells decreased sensitivity to AgNP, and knockdown of neurofibromin in isogenic Schwann cells increased sensitivity to AgNP, outlining a correlation between neurofibromin expression and AgNP-mediated cytotoxicity. AgNP was able to selectively remove pNF cells from a co-culture with patient-matched Schwann cells. Therefore, AgNPs represent a new approach for clinical management of NF1-associated pNF to address significant clinical need.

## 1. Introduction

Neurofibromatosis type 1 (NF1) is one of the most common neurogenic diseases. NF1 is an autosomal dominant disease that affects around 1:3000 people and is characterized by loss of function mutations in the neurofibromin gene (*NF1*) [1,2]. NF1 is a multisystem disease affecting various body systems. Clinical manifestations include café au lait spots, freckling, Lisch nodules, gliomas, and cognitive deficiencies noted by problems with behavior, learning, and social adaptation [1]. NF1 patients also develop neurofibromas with near-complete penetrance [3]. Unfortunately, NF1 patients have a life expectancy 10–15 years shorter than the general population [4], thought to be at least in part due to increased risk of cancers with a poorer overall prognosis [5].

NF1 patients are predisposed to the development of peripheral nerve sheath tumors. A plexiform neurofibroma (pNF) is a rare type of nerve sheath tumor, which is typically associated with NF1, that arises from Schwann cells in peripheral nerves [6]. Plexiform neurofibromas are characterized by their diffuse and plexiform (network-like) growth pattern, which distinguishes them from other types of neurofibromas. These tumors can occur at any age, but are most commonly diagnosed in children [2]. They can affect various parts of the body, including the limbs, face, trunk, and internal organs. Plexiform neurofibromas can be large and disfiguring, causing cosmetic concerns and functional impairments. The symptoms of plexiform neurofibromas vary depending on their location and size. They may cause pain, weakness, numbness, or loss of function in the affected area [6]. Additionally, plexiform neurofibromas carry a risk of malignant transformation into malignant peripheral nerve sheath tumors (MPNSTs) [7], which are an aggressive form of cancer and represent the most common cause of death in NF1 patients [8].

Plexiform neurofibroma formation is initiated and promoted by complete loss of functional neurofibromin expression [9]. Neurofibromin is a tumor suppressor having a well-characterized RasGAP-related domain, which increases the hydrolysis rate of Ras-GTP to the inactive Ras-GDP form [10]. Neurofibromin is a key regulator which suppresses Ras activity under normal circumstances. In non-cancerous cells, Ras integrates external signaling to intracellular processes, to promote normal cell processes such as differentiation, survival, and proliferation [11]. However, inappropriately sustained Ras activity, such as that seen in pNF, drives tumorigenesis and progression [9], disrupts redox balance which can alter metabolism, increases reactive oxygen species, and ultimately increases overall oxidative stresses [12].

The standard of care treatment for plexiform neurofibroma is surgical excision based on location and accessibility [6]. Inoperable pNF are treated with MEK inhibitor selumetinib, which was FDA approved in 2020 [13]. However, selumetinib showed variable penetrance evidenced by an overall response rate of 66%, and only 82% of patients achieving a duration of response greater than one year [14]. Follow-up studies showed that 72% of patients achieved a confirmed partial response, defined as a pNF volume decrease of 20% or greater, on consecutive scans at least three months apart [15]. Selumetinib is useful for the treatment of inoperable pNF; however, it is now evident that not all pNF respond to selumetinib and off-target effects can hamper utility. Thus, additional methods to treat inoperable pNF should be evaluated.

Nanomedicine shows promise as a novel treatment for cancer, including plexiform neurofibroma. Silver metal-based therapies in particular show potential in the development of novel cancer therapeutics. Silver metal formulated as silver nanoparticles (AgNPs) are cytotoxic to a variety of cancer cell types derived from an array of tissues that have minimal effect on normal cells [16,17,18,19,20,21,22,23,24]. We previously optimized an AgNP, which consists of silver metal (Ag^0^) and FDA-safe excipient polyvinylpyrrolidone (PVP, 40 kDa), that is safe for long-term systemic administration in murine models, and shows evidence of efficacy against triple-negative breast cancer in vivo [21]. The AgNPs used in this study and in our previous work are commercially available, 25 nm in diameter, have a silver content of 15% by weight and were chosen based on existing data and tolerable pharmacokinetic properties [19,20,21]. Mechanistically, we found that this particular formulation of AgNPs induce an array of cellular effects including: DNA damage [19,21]; protein oxidation; mitochondrial stress [24]; lipid peroxidation [20,25]; disrupted redox balance; and overall increased oxidative stress [22,25].

AgNP-mediated cytotoxicity relies upon increased basal oxidative stress, such as that promoted by the inappropriately sustained Ras activity seen in NF1-associated pNF. Zero valent silver (Ag^0^ as found in AgNPs) is non-toxic, but can be rapidly ionized in cancer cells to silver ion (Ag^+^) as cancer cells have increased basal oxidative stress [21,26]. Ag^+^ is known to be extremely cytotoxic; however, normal cells do not appreciably ionize Ag^0^. This cancer-specific activation (through ionization) is the basis for the cancer-selective AgNP-mediated cytotoxicity which does not affect normal healthy cells. Importantly, the formulation of AgNP used in this study does not release silver ion during storage [19] and can be safely administered systemically [21].

Our recent study also found that AgNPs show preclinical efficacy for the treatment of NF1-associated MPNSTs, which can arise from pNF after transformation [18]. We further discovered that AgNP-mediated cytotoxicity is dependent on loss of functional neurofibromin expression. Loss of neurofibromin expression leads to inappropriate RAS activity and increased basal oxidative stress, and is an initiating step in the development of pNF. These data provide support for the evaluation of AgNP as a treatment for neurofibromin-deficient pNF. Therefore, we hypothesized that we could exploit this cancer-selective activation, and develop a novel therapy for neurofibromin-deficient pNF to complement current therapies. In this study, we evaluated AgNPs as a cancer-selective treatment for pNF, and the role of neurofibromin expression in AgNP-mediated cytotoxicity, using an NF1-patient-matched plexiform neurofibroma and Schwann cell in vitro cell model system.

## 2. Materials and Methods

### 2.1. Cell Culture

Immortalized cell lines ipNF95.11bC (plexiform neurofibroma) and ipnNF95.11c (NF1 patient-matched Schwann cells) were generously provided by Margaret Wallace (University of Florida, Gainesville, FL, USA), are available in public repositories, and are further detailed in [27]. The necessary amount of 293T cells (CRL-3216) were purchased from American Type Culture Collection. All cells used in this study were tested for mycoplasma contamination using luciferase-based monitoring (MP Biomedicals, Seven Hills, Australia), and were used within 15 passages from cryo-resurrection. Cells were passaged 2–3 times per week and maintained in complete medium, which consisted of Dulbecco’s Modified Eagle Medium (DMEM, Gibco, Waltham, MA, USA) containing 10% fetal bovine serum (GemCell (USA Origin)); GeminBio, West Sacramento, CA, USA); penicillin (250 units mL^−1^); streptomycin (250 μg mL^−1^); and L-glutamine (2 mmol/L) (Gibco), at 37 °C/5% CO_2_ environmental conditions.

### 2.2. Silver Nanoparticles

Silver nanoparticles (AgNPs) were purchased from Nanocomposix, Inc. (San Diego, CA, USA). The AgNPs used in this study are spherical in shape, 25 nm in diameter, and are stabilized with polyvinylpyrrolidone (40 kDa) with a 15% silver content by weight. AgNPs were stored as a dry powder in a desiccator cabinet protected from light. Prior to use AgNPs were dispersed in physiological solution (phosphate-buffered saline (PBS), pH 7.4, without calcium or magnesium), with the aid of an ultrasonic bath at a final concentration of 5 µg mL^−1^ Ag. AgNPs in suspension were stored at 4 °C, protected from light, and used within one month of initial dispersion. AgNPs were serially diluted in complete medium immediately prior to treatment in all experiments described. AgNPs were previously characterized (long-term colloidal stability in physiological solution, zeta potential, hydrodynamic diameter) by Nanocomposix and in [19,21].

### 2.3. Zeta Potential

Zeta potential was measured using a Zetasizer Nano ZS90 (Malvern Instruments, Worcestershire, UK) in a disposable folded capillary cell (Malvern Instruments). Zeta potential was measured in triplicate at a concentration of 250 µg/mL in deionized water at 25 °C.

### 2.4. Real-Time Quantitative PCR

Neurofibromin RNA transcript levels were quantified via real-time quantitative PCR. RNA was extracted using TRIZOL reagent (Invitrogen, Waltham, MA, USA) with the aid of Direct-zol RNA kit (Zymo Research, Irvine, CA, USA) according to the manufacturer’s protocol, including DNAse treatment to remove any residual genomic DNA. Specifically, confluent plates of cells (2.5–3.0 × 10^6^ cells, 60 mm TC dish (Sarstedt, Newton, NC, USA)) were harvested in 1 mL TRIZOL reagent, followed by RNA isolation. Extracted RNA quantity and quality was determined using a DeNovix DS-11+ spectrophotometer (Wilmington, DE, USA). RNA absorbance was measured at 230, 260, and 280 nm. All RNA samples were verified to have a 260/230 ratio between 1.8 and 3, and a 260/280 ratio between 1.65 and 2.5, before cDNA synthesis and real-time quantitative PCR. Copy DNA (cDNA) was prepared using a High-Capacity cDNA Reverse Transcription Kit with RNase Inhibitor kit (Applied Biosystems, Norwalk, CT, USA) with 1.0 μg RNA input in a GeneAmp PCR System 9700 (Applied Biosystems). Taqman reagents specific for neurofibromin (Hs01035108_m1) and reference gene PPIA (Hs04194521_s1, validated in [28] as an optimal reference gene for benign Schwann cell lines) were purchased from Applied Biosystems. Realtime quantitative PCR was performed using Taqman Fast Advanced Master Mix in quadruplicate using a 384-well format in a QuantStudio 6 Flex (Applied Biosystems). ∆∆Ct methodology was used to calculate relative levels of neurofibromin across samples as detailed in [18].

### 2.5. Viability Assays

#### 2.5.1. MTT Assay

Cell viability was determined using 3-(4,5-Dimethylthiazol-2-yl)-2,5-diphenyltetrazolium bromide (MTT) reagent from VWR. MTT assay reagent was prepared in PBS at a final concentration of 5 mg mL^−1^, filter sterilized, stored protected from light at 4 °C, and used within two weeks of preparation. Plexiform neurofibroma cells or patient-matched Schwann cells were seeded in 96-well culture dishes (6.0 × 10^3^ cells in 100 μL complete growth medium). The following day cells were exposed to AgNP or selumetinib through addition of 100 μL of 2× treatment to each well in quintuplicate. After 72 h incubation, 20 μL of prepared MTT reagent was added to each well (including a cell-free control well), and incubated for 75–90 min at 37 °C/5% CO_2_, as performed in [19,20,21,22,25]. Medium was removed and 150 μL dimethyl sulfoxide was added. Contents of each well were gently mixed using a pipet. Viability was determined by measuring absorbance at 560 nm. Readings were background-corrected using absorbance at 650 nm. Treated cells were compared to untreated cells to determine viability. All measurements were performed using a Spectramax iD5 plate reader.

#### 2.5.2. ATP Assay

Cell viability was determined using CellTiter-Glo luminescent assay (Promega, Madison, WI, USA). CellTiter-Glo was prepared and stored as detailed in the manufacturer’s protocol. Plexiform neurofibroma cells or patient-matched Schwann cells were seeded in 96-well culture dishes (6.0 × 10^4^ cells in 100 μL complete growth medium). The following day, cells were exposed to AgNP through addition of 100 μL of 2× treatment to each well in triplicate, and incubated for 72 h at 37 °C/5%. Wells were gently aspirated, and 75 μL PBS and 75 μL CellTiter-Glo was added. Plates were incubated for 3 min on an orbital shaker at 300 RPM. Lysates were transferred to an Eppendorf tube and centrifuged (500× *g*, 5 min) to remove any remaining intact silver nanoparticles. Cleared lysate (100 μL) was transferred to a white 96-well plate and total luminescence measured using a Spectramax iD5 plate reader.

#### 2.5.3. Trypan Blue Exclusion

Cell viability was determined using trypan blue and counting with a hemacytometer. Plexiform neurofibroma cells or patient-matched Schwann cells were seeded in 24-well culture dishes (3.5 × 10^4^ cells in a volume of 0.6 mL per well). Seeding density per cm^2^ was consistent with MTT and ATP viability assays detailed above. The following day, cells were treated with AgNP through addition of 600 μL 2× treatment to each well in triplicate. After 72 h, cells were gently washed in PBS, and 100 μL trypsin (Corning, Corning, NY, USA) was added to detach cells. Complete medium (900 µL) was added to neutralize trypsin. Cell suspension was mixed with Trypan Blue (Corning) in a 1:1 ratio. Total number of trypan blue excluded cells (viable) were counted for each well using a hemacytometer.

### 2.6. Lentivirus Production

Lentiviral particles were prepared using 293T cells, as detailed in [18]. Envelope plasmid psPAX (#12259); packaging plasmid pMD2.G (#12260); dsRed2-RFP expression vector (#109377); and GFP expression vector (#17448) were purchased from Addgene. NF1-GFP expression vector was generously provided by Robert F. Hennigan (University of Cincinnati, Cincinnati, OH, USA). NF1-GFP viral supernatant was further concentrated with Lenti-Pac Lentivirus Concentration Solution per the manufacturer’s protocol. Lentiviral supernatants were mixed with Lenti-Pac Concentration Solution in 4:1 ratio and incubated on ice for 18 h, then the mixture was centrifuged (3500× *g* for 5 min at 4 °C), supernatant aspirated, and lentiviral pellet resuspended in a volume 1100 of original starting volume. Concentrated lentiviral supernatant and concentrated lentivirus was aliquoted for single-use and stored at −80 °C.

### 2.7. Cell Transduction

The ipnNF95.11C and ipNF95.11bC cells (2.5 × 10^5^ cells per well in 2.5 mL complete medium) were seeded in 6-well tissue culture plates (VWR) and allowed to attach for 18 h. Medium was aspirated and either 0.5 mL viral supernatant (shRNA constructs, GFP, RFP) and 0.5 mL complete medium or 25 μL concentrated viral supernatant (NF1-GFP) and 975 μL complete medium was added to each well. Cells were incubated under standard conditions for 24 h. Cells were then puromycin selected at a concentration of 2 μg mL^−1^. After selection was complete through visual comparison to a non-transduced control, pooled clones were grown in complete medium and used for the indicated studies.

### 2.8. Co-Culture Model System

ipnNF95.11C-RFP (9.0 × 10^4^ cells) and ipNF95.11bC-GFP (9.0 × 10^4^ cells) were seeded together as a 1:1 co-culture in a 6-well format and allowed to attach overnight as in [18]. Seeding density per cm^2^ was consistent with viability assays detailed above. Cells were then treated with AgNP (0–25 μg mL^−1^) or vehicle (PBS) in a total volume of 3 mL complete culture medium for 48 h. For imaging, wells were gently aspirated and washed once with Live Cell Imaging Solution (Gibco). During imaging cells remained in fresh Live Cell Imaging Solution for a period less than 30 min. Cells were imaged with an EVOS FL Microscope (Life Technologies, Carlsbad, SD, USA) with 10× magnification. Each well was imaged using transmitted light, and LED cubes specific for GFP and RFP. The ipnNF95.11C-RFP or ipNF95.11bC-GFP were seeded (1.8 × 10^5^ cells in 2.5 mL complete medium) in triplicate in a 6-well format as a monoculture. Cells were treated as above for mono-culture viability assays using MTT assay for 48 h as above, except with the addition of 300 μL prepared MTT reagent to each well and resuspending in a total volume of 1.5 mL dimethyl sulfoxide.

### 2.9. Other Reagents

Selumetinib was purchased from Selleck Chemical (Houston, TX, USA); silver nitrate was manufactured by Acros (Geel, Belgium).

### 2.10. Statistical Analysis

Graphpad Prism 9 was used to calculate IC_50_ of AgNP and selumetinib for the indicated cell models, using variable slope with least squares methodology. Statistical significance was determined by comparing these IC_50_ values between cell lines using a two-tailed student’s *t*-test assuming equal variance. Statistical significance comparing cell viability assays was also determined using a two-tailed student’s *t*-test assuming equal variance Significance is indicated in this study using the following: ***, *p* < 0.001; **, *p* < 0.01; *, *p* < 0.05; n/s, *p* > 0.05.

## 3. Results

### 3.1. Physiochemical Characterization of Silver Nanoparticles

**Figure 1:**
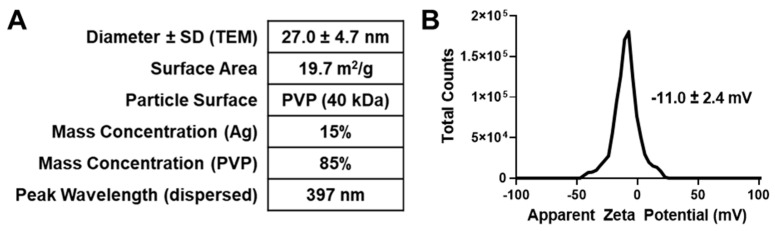
Physiochemical characterization of silver nanoparticles. (**A**) Silver nanoparticles (AgNPs) were purchased in the form of dried powder from Nanocomposix. Key physiochemical characteristics provided by manufacturer (Nanocomposix) are shown. (**B**) Zeta potential was measured with AgNPs dispersed in water at a concentration of 250 µg mL^−1^, and is shown ± SD as inset in graph.

Silver nanoparticles (AgNPs), 25 nm in diameter and stabilized with polyvinylpyrrolidone (PVP, 40 kDa), were purchased in the form of dried powder from Nanocomposix. Manufacturer-provided characterization showed that the AgNPs were 27.0 ± 4.7 nm in diameter (using transmission electron microscopy). The AgNPs used in this study had a percent mass of Ag of 15%, as determined via ICP-MS. Further characterization performed by the manufacturer is shown in Figure 1A. We determined the zeta potential to be 11.0 ± 2.4 mV (Figure 1B). We have previously characterized this particular nanoparticle formulation and found them to be colloidally stable after hydration in water, saline, medium, and serum for extended periods of time [21,25]. This formulation also shows a tolerable safety profile in murine in in vivo models [21]. Based on these characteristics, this formulation of AgNPs is the most suitable lead formulation for preclinical evaluation of a novel treatment for pNF.

### 3.2. Silver Nanoparticles Are Selectively Cytotoxic to Plexiform Neurofibroma

Plexiform neurofibromas most often develop in patients with NF1 after complete loss of neurofibromin, and are thought to be of Schwann cell origin. Our previous study showed that AgNPs are selectively cytotoxic to NF1-associated MPNSTs in a neurofibromin-dependent manner [18], thus we hypothesized that AgNP may be a rational novel treatment for neurofibromin-null plexiform neurofibroma. In order to test the effectiveness of AgNPs as a treatment for pNF, we used a patient-matched in vitro cell model system. Cells ipNF95.11bC are immortalized plexiform neurofibroma cells derived from a NF1 patient which lack functional neurofibromin, and ipnNF95.11C are patient-matched Schwann cells harboring a single functional copy of neurofibromin [27]. This is the most appropriate model for preclinical development of novel therapies targeted to NF1 patients thus allowing us to test the efficacy of AgNP as a treatment for NF1-related pNFs with an isogenic normal cell control.

**Figure 2:**
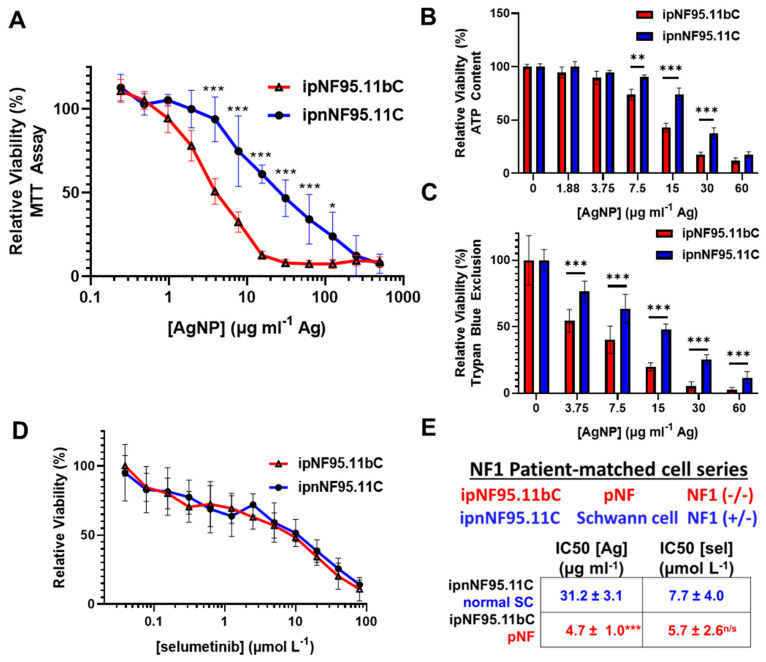
AgNP show preferential cytotoxicity in plexiform neurofibromas cells compared to NF1 patient-matched Schwann cells. NF1-associated plexiform neurofibroma cells (ipNF95.11bC) and patient-matched normal Schwann cells (ipnNF95.11C) were treated with AgNPs for 72 h, and viability was evaluated via (**A**) MTT viability assay, (**B**) ATP-based CellTiter Glo assay, and (**C**) trypan-blue exclusion. (**D**) Plexiform neurofibroma and patient-matched cells were treated with standard-of-care selumetinib for 72 h and viability evaluated via MTT assay; no significant difference exists between cell lines (**E**). Cell line *NF1* status, and IC_50_ for AgNP and selumetinib derived from MTT assays is shown by μg mL^−1^ Ag, or μmol L^−1^ selumetinib ± SEM. MTT assay data represents four independent experiments per cell line each containing five technical replicates. Significance between IC_50_ and viability between cell lines was determined via Student’s *t*-test (* *p* < 0.05, ** *p* < 0.01, *** *p* < 0.005, n/s not significant).

Plexiform neurofibroma cells (ipNF95.11bC) and NF1-patient matched Schwann cells were treated with AgNP ranging from 0–500 μg mL^−1^ (by Ag content) for 72 h. AgNPs were significantly more cytotoxic to ipNF95.11bC relative to isogenic control cell line ipnNF95.11C, as determined via MTT viability assay (Figure 2A). Similarly significant results were found when viability was assessed by both ATP content (Figure 2B) and trypan-blue exclusion (Figure 2C). The standard-of-care treatment for non-resectable pNF is MEK inhibitor selumetinib. Therefore, we tested our cell panel with standard-of-care therapy selumetinib (0–80 μmol L^−1^) for 72 h. In stark contrast, there was no clear delineation in selumetinib sensitivity comparing pNF cells and patient-matched Schwann cells (Figure 2D). AgNP IC_50_ values were calculated to be 3.1 ± 1.0 μg mL^−1^ Ag and 31.2 ± 3.0 μg mL^−1^ Ag, respectively (Figure 2E). These data suggest that AgNP could improve the clinical management of inoperable plexiform neurofibroma compared to standard-of-care therapy.

### 3.3. Intact Silver Nanoparticles Are Required for Plexiform Neurofibroma Selectivity

**Figure 3:**
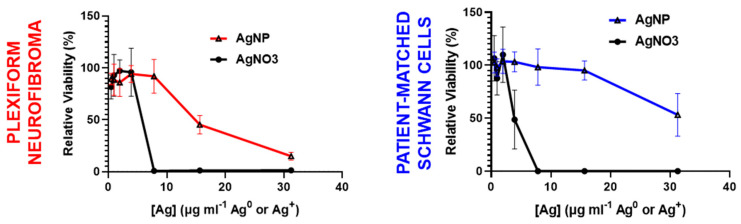
Intact AgNPs are required for plexiform neurofibroma-selective cytotoxicity. Plexiform neurofibroma cells (ipNF95.11bc) (**left panel**) and patient-matched Schwann cells (ipNF95.11C) (**right panel**) were exposed to AgNPs (intact silver nanoparticles, Ag^0^) or AgNO_3_ (silver ion, Ag^+^) at equivalent silver doses for 48 h and viability assessed via MTT. Data is shown ± SD and is representative of three independent experiments.

Our previous studies show that AgNP-mediated cytotoxicity occurs after rapid oxidation of intact AgNPs (Ag^0^) into silver ion (Ag^+^) in triple-negative breast cancer, relative to slower oxidation in normal breast epithelial cells [21]. Therefore, we evaluated whether Ag^0^ or Ag^+^ is responsible for plexiform neurofibroma selective cytotoxicity, as cancer selectivity is crucial for preclinical development. To test this hypothesis, we treated pNF cells (ipNF95.11bC) and patient-matched Schwann cells (ipnNF95.11C) with equivalent silver, using AgNP as a source of Ag^0^ and silver nitrate as a source of Ag^+^. We found that both pNF cells and patient-matched Schwann cells were similarly sensitive to Ag^+^, but showed variable sensitivity to equivalent mass of silver in the form of Ag^0^ (Figure 3). These data demonstrate that pNF-selectivity requires silver in the form of Ag^0^ and support the requirement of this particular nanoparticle formulation for the selective treatment of plexiform neurofibroma.

### 3.4. Restoration of Functional Neurofibromin Expression in Plexiform Neurofibroma Decreases Sensitivity to Silver Nanoparticles

**Figure 4:**
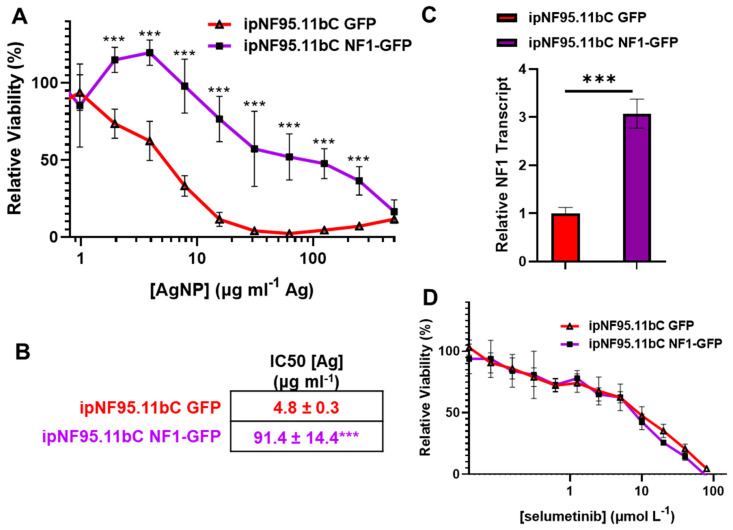
Reestablishment of functional neurofibromin expression reduces AgNP-mediated cytotoxicity in plexiform neurofibroma cells. The ipNF95.11bC plexiform neurofibroma cells were transduced with plasmids containing NF1-GFP or GFP as control, selected with puromycin, and clones pooled. (**A**) ipNF95.11bC-NF1-GFP and ipNF95.11bC-GFP or were treated with AgNP (0–500 μg mL^−1^ Ag) for 72 h, and viability determined via MTT assay. (**B**) IC_50_ values for AgNP are shown ± SEM. (**C**) qPCR specific for NF1 and the housekeeping gene PPIA was performed in quadruplicate, and relative neurofibromin transcript levels were calculated using ΔΔCT methodology and are shown ± SD. (**D**) Cells were treated with standard-of-care selumetinib (0–80 μmol mL^−1^) for 72 h, and viability assessed via MTT assay; no significant difference was found between cell lines at all tested concentrations. Viability data represent four independent experiments per cell line each containing five technical replicates. Significance between cell lines was determined via Student’s *t*-test (*** *p* < 0.005).

Neurofibromin is a negative regulator of Ras signaling. Loss of neurofibromin is known to increase reactive oxygen species and intracellular oxidative stress [29] which potentiates AgNP ionization/activation [21], and is a major driver in the development of plexiform neurofibroma. Biallelic loss of neurofibromin is a key difference between our plexiform neurofibroma cell model and the patient-matched Schwann cell control cell model. Since our previous study shows that functional neurofibromin expression has a direct effect on AgNP sensitivity in MPNSTs [18], we hypothesized that functional neurofibromin expression would also impact AgNP-mediated cytotoxicity in plexiform neurofibroma, as they are thought to ultimately transform into MPNSTs. To evaluate this hypothesis, we transduced our cell model of plexiform neurofibroma (ipNF95.11bC) cells with an expression vector containing functional neurofibromin tagged with green fluorescent protein, or an expression vector which contained green fluorescent protein to be used as control. Following puromycin selection, pooled clones of transfected cells (termed ipNF95.11bC-NF1-GFP and ipNF95.11bC-GFP) were treated with AgNP 0–500 μg mL^−1^ (by Ag content) for 72 h. The ipNF95.11bC-NF1-GFP cell line was significantly less susceptible to AgNP-mediated cytotoxicity relative to the control ipNF95.11bC-GFP, with IC_50_ values calculated to be 91.4 ± 14.4 μg mL^−1^ Ag and 4.8 ± 0.3 μg mL^−1^ Ag, respectively (Figure 4A,B). Levels of relative neurofibromin expression were determined using previously validated probes (Figure 4C). We then treated ipNF95.11bC-NF1-GFP and ipNF95.11bC-GFP with standard-of-care selumetinib and found there was no change in sensitivity to selumetinib (Figure 4D). These data support the direct link between functional neurofibromin expression and sensitivity to AgNP, whereas a direct link is not apparent between functional neurofibromin expression and sensitivity to selumetinib.

### 3.5. Knockdown of Neurofibromin Increases Silver Nanoparticle Sensitivity in NF1-PatientDerived Schwann Cells

**Figure 5:**
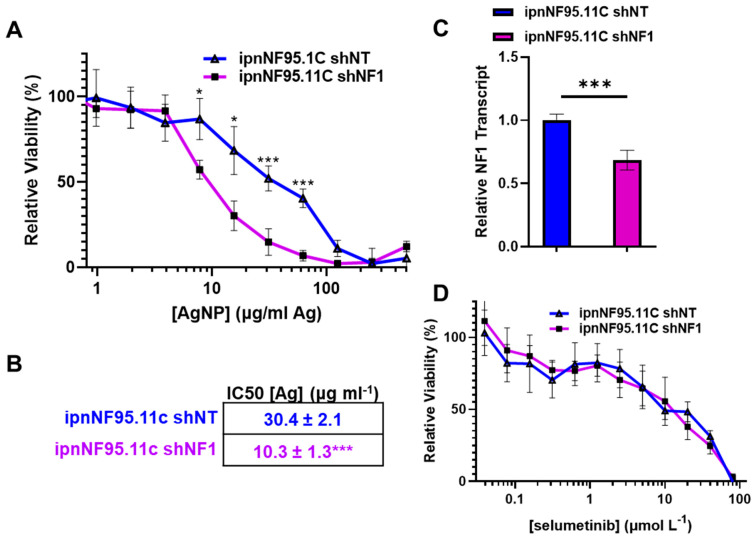
Decreasing neurofibromin expression increases sensitivity to silver nanoparticles in NF1-patient-derived Schwann cells. The ipnNF95.11C NF1-patient-derived Schwann cells were transduced with lentiviral particles containing shRNA against NF1 (shNF1) or non-targeted control (shNT), puromycin selected and clones pooled. (**A**) ipnNF95.11C-shNF1 or control ipnNF85.11C-shNT were AgNP-treated (0–500 μg mL^−1^ Ag) for 72 h, and viability determined using MTT assay. (**B**) IC_50_ values for AgNP are shown ± SEM. (**C**) qPCR specific for NF1 and the housekeeping gene PPIA was performed in quadruplicate, and relative neurofibromin transcript levels were calculated using ΔΔCT methodology and are shown ± SD. (**D**) Cells were treated with selumetinib (0–80 μmol mL^−1^) for 72 h, and viability assessed via MTT assay; no significant difference was found between cell lines at all tested concentrations. Data represent four independent experiments per cell line, each containing five technical replicates Significance between cell lines was determined via Student’s *t*-test (* *p* < 0.05, *** *p* < 0.005).

NF1 patients are much more susceptible to developing pNFs, as NF1 diagnosis itself requires a heterozygous neurofibromin pathogenic variant. Specifically, in NF1 patients, pNFs develop after complete loss of functional neurofibromin. Based on the finding that restoration of functional neurofibromin decreases sensitivity to AgNP, we sought to evaluate the consequence of loss of functional neurofibromin in NF1-patient-derived Schwann cells as it relates to AgNP-mediated cytotoxicity. Knockdown of neurofibromin was performed as detailed above and in [4], using ipnNF95.11C as a model of NF1-associated Schwann cells. We found that reducing expression of neurofibromin alone is sufficient to increase sensitivity to AgNP in NF1-patient-derived Schwann cells (Figure 5A), with IC_50_ values calculated to be 30.4 ± 2.1 μg mL^−1^ Ag for ipnNF95.11C—shNT and 10.3 ± 1.3 μg mL^−1^ Ag for ipnNF95.11C—shNF1. Knockdown of neurofibromin transcript was confirmed using quantitative PCR (Figure 5C). We then challenged ipnNF95.11C-shNF1 with standard-of-care selumetinib and found that decreased expression of neurofibromin did not affect sensitivity (Figure 5D). Our evidence further supports the correlation between functional neurofibromin expression and sensitivity to AgNP in pNFs and an in vitro model of tumor initiation.

### 3.6. Silver Nanoparticles Selectively Remove NF1-Associated Plexiform Neurofibromin Cells from Co-Culture with Patient-Matched Schwann Cells

A therapeutic window must exist for the successful development of novel therapeutics, as tumors contain multitudes of cell types including cancerous and normal healthy cells. Successful treatment ultimately relies on the ability to safely remove cancerous cells while leaving healthy cells unharmed. To further evaluate the preclinical efficacy of AgNP as a treatment for pNF, we developed an in vitro co-culture in which we could distinguish pNF cells from patient-matched Schwann cells. We stably transduced our cell models to express fluorescent proteins to follow each cell type. Using lentiviral transduction, we engineered pNF cells and patient-matched Schwann cells to express green fluorescent protein and red fluorescent protein, respectively. These labeled cells were termed ipNF95.11bC-GFP (pNF), and ipnNF95.11C-RFP (patient-matched isogenic Schwann cells).

**Figure 6:**
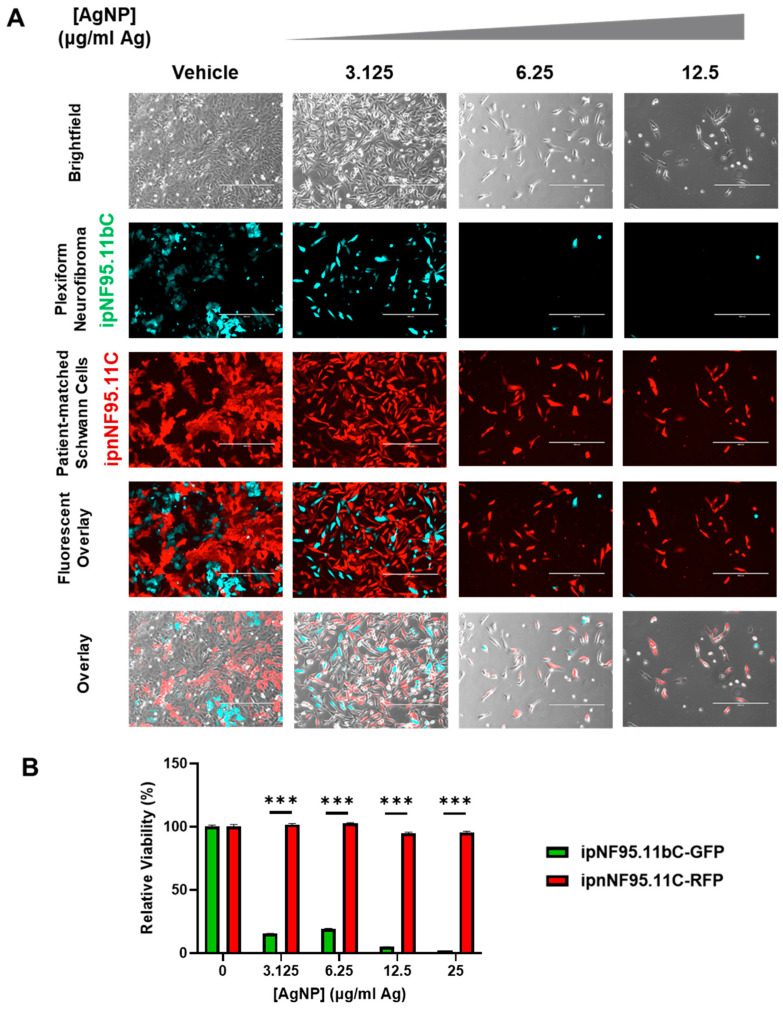
AgNP selectively removes plexiform neurofibroma cells from co-culture with patient-matched Schwann cells. Plexiform neurofibroma cells (ipNF95.11bC) and patient-matched control cells (ipnNF95.11C) were fluorescently labeled using lentiviral transduction, selected with puromycin, and clones were pooled. The ipNF95.11bC and ipNF95.11C were labeled as RFP and GFP, respectively. (**A**) ipnNF95.11bC-GFP and ipNF95.11C-RFP were seeded at a 1:1 ratio and treated with AgNP (0–12.5 μg mL^−1^ Ag) for 48 h. Representative images are shown after continuous treatment with AgNP. (**B**) ipnNF95.11C-RFP and ipNF95.11bC-GFP were grown under the same conditions as (**A**) as monocultures and treated with AgNP (0–25.0 μg mL^−1^) for 48 h, and viability assessed via MTT assay. Data is representative of three independent experiments each containing four technical replicates. Significance between cell lines was determined via Student’s *t*-test (*** *p* < 0.005).

An equal number ipNF95.11bC-GFP and ipnNF95.11C-RFP were grown in the same culture vessel and treated with AgNP for up to 48 h. Microscopy showed that AgNP were able to remove pNF cells at doses that allowed patient-matched Schwann cells to survive (Figure 6A). To ensure that ipnNF95.11C-RFP were still viable at the doses tested, companion monocultures were performed under the same conditions and viability was assessed via MTT. The remaining cells were found to be viable (Figure 6B). It is clear in the co-cultures that the death of ipNF95.11bC-GFP partially impacted the viability of ipnNF95.11c-RFP when in co-culture, which could be due to the shared culture medium containing an amalgam of cellular components and dying cells. Nonetheless, this model system and evidence support the existence of a therapeutic window to use AgNP in the clinical management of NF1-associated plexiform neurofibromas.

## 4. Discussion

**Figure 7:**
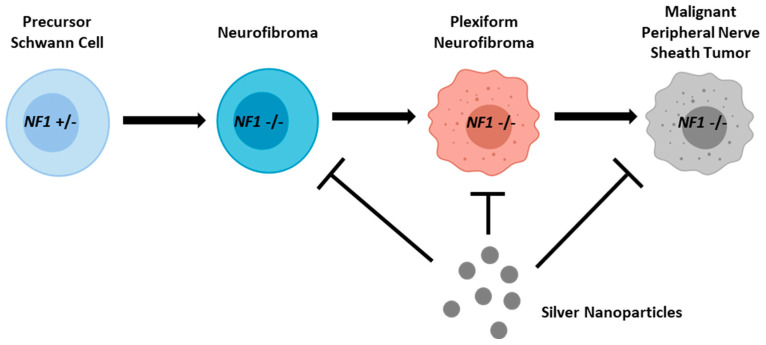
Silver nanoparticles show promise in the clinical management of NF1-associated neurofibroma. Precursor Schwann cells undergo oncogenesis after biallelic loss of neurofibromin and form neurofibromas. These neurofibromas can further develop into problematic benign neurofibromas (including plexiform neurofibromas), which can undergo malignant transformation into deadly malignant peripheral nerve sheath tumors. AgNPs are selectively cytotoxic to NF1-associated MPNSTs and plexiform in a neurofibromin dependent manner compared to both NF1 wild-type Schwann cells and NF1-patient-derived Schwann cells (Figure 2 and [18]). AgNP-mediated cytotoxicity is potentiated upon loss of neurofibromin. Knockdown of neurofibromin in NF1-patient-derived Schwann cells is sufficient to confer sensitivity to AgNP. Therefore, AgNP may be useful in the treatment of established pNFs which do not respond to selumetinib; pNF progenitor Schwann cells which have undergone biallelic loss of neurofibromin; and malignant peripheral nerve sheath tumors [18].

Plexiform neurofibroma are peripheral nerve sheath tumors that occur in half of all NF1 patients after biallelic loss of the neurofibromin gene. Successful treatment of pNF is crucial as pNF itself can cause significant debilitation and pain, but can also malignantly transform into MPNSTs, which is the most common cause of death in NF1 patients [8]. Non-resectable pNFs are treated with the MEK inhibitor selumetinib, which can lack a complete durable response and dose-limiting off-target effects [15]. Here we show that our simple two-component silver nanoparticle formulation (silver metal (Ag^0^) and polyvinylpyrrolidone-coating) shows preferential cytotoxicity in pNF cells compared to isogenic Schwann cells in a neurofibromin expression-dependent manner and are able to selectively remove pNF cells from a co-culture in and in vitro model system. Together, these data support preclinical efficacy of AgNP for the treatment of plexiform neurofibroma which lacks expression of functional neurofibromin (Figure 7).

Our group and others show that AgNPs show cytotoxicity to cancer cells of a variety of tissue origins at doses that have minimal effect on normal cell models [16,17,18,19,20,21,22,23,24]. Recently, we showed that AgNPs show selectivity in NF1-associated MPNSTs relative to tumor cell-of-origin Schwann cells in a functional neurofibromin-expression dependent manner [18]. To the best of our knowledge, we are the first group to show evidence supporting the preclinical efficacy of AgNP for the treatment of NF1-associated pNF. In this study, we use NF1-patient matched cell lines derived from both plexiform neurofibroma and adjacent Schwann cells. It is crucial to use NF1-patient-derived cells as control which are heterozygous for neurofibromin, as functional neurofibromin expression directly impacts sensitivity to AgNPs (Figure 4 and Figure 5, and in [18]). This model system allows for evaluation of our AgNP using an in vitro model that best represents what is seen in NF1 patients, while maintaining an isogenic background for clinical relevance.

AgNPs are known to have pleiotropic effects in cancer cells of disparate origin such as protein oxidation [20]; mitochondrial stress [24]; lipid peroxidation [25]; endoplasmic reticulum stress [21]; and DNA damage [19,21,22]. AgNPs have been shown to induce these catastrophic damages in mesenchymal-like cancers, which include pNFs. Based on these previous findings it is likely that pNF cells will be affected in a similar manner and warrants mechanistic studies in the context of plexiform neurofibroma.

Neoplastic progression of Schwann cells into pNF after loss of neurofibromin includes activation of genes associated with a mesenchymal-like transition [30,31], and an increase in basal oxidative stress through potentiated Ras activity [29]. AgNP-mediated cytotoxicity in mesenchymal-like cancer-specific effects is due to rapid activation through ionization of non-toxic Ag^0^ into cytotoxic Ag^+^, which occurs at a much more rapid rate in cancer cells relative to normal cells [21]. Development of pNF is characterized by a complete loss of functional neurofibromin, which in turn may increase basal oxidative stress in such a way that imparts sensitivity to AgNP through preferential ionization of silver metal into cytotoxic silver ion in pNF cells, relative to normal Schwann cells.

A significant finding in this study is the correlation between functional neurofibromin expression and sensitivity to silver. Plexiform neurofibroma cell line ipNF95.11bC is significantly more sensitive to AgNP compared to NF1-patient-matched control ipnNF95.11C Schwann cells. We noted that restoration of functional neurofibromin in ipNF95.11bC reduced sensitivity to AgNP (Figure 4A). Conversely, knockdown of neurofibromin ipnNF95.11C increased sensitivity to AgNP (Figure 5A). This finding is unique to AgNP as there was no evidence of changes in sensitivity to standard-of-care selumetinib. These data show that expression of a single gene, in this case neurofibromin, is directly correlated with AgNP-mediated cytotoxicity. We propose that lack of functional neurofibromin expression may be a useful biomarker for the rational use of AgNP as a treatment for peripheral nerve sheath tumors including pNF. Clearly, more investigation is necessary to establish the mechanism by which neurofibromin expression directly alters sensitivity to AgNP.

Our previous studies also support the direct role of functional neurofibromin expression on AgNP-mediated sensitivity in NF1-associated peripheral nerve sheath tumors. We found that NF1-associated MPNSTs, which lack functional neurofibromin, were much more sensitive to AgNP compared to Schwann cells, which harbored two functional copies of *NF1* [18]. This study showed that pNF cells which also lack functional neurofibromin were much more sensitive to AgNP compared to NF1-patient-matched Schwann cells, which only harbor a single functional copy of *NF1*. These findings demonstrate that the gene dose of functional neurofibromin is a major determinant of AgNP sensitivity and a possible biomarker for precision medicine use of AgNP. Ranked from most AgNP-sensitive to least AgNP-sensitive are NF1-associated MPNSTs (*NF1*^−/−^); NF1-associated plexiform neurofibroma (*NF1*^−/−^); NF1-associated Schwann cells (*NF1*^+/−^); and Schwann cells (*NF1^+/+^*), which also corresponds to the increasing gene dose of functional neurofibromin. It is interesting to note that even a partial restoration of functional neurofibromin is sufficient to “revert” ipNF95.11bC to an AgNP sensitivity similar to that of isogenic Schwann cell ipnNF95.11C (Figure 4A) and in normal Schwann cells [18].

This well-established neurofibromin genetic switch in pNF is the key to the development of AgNP as a potential treatment for an inoperable pNF that does not respond to selumetinib. Cancer-selective activation of AgNPs would allow for systemic administration and there is evidence which suggests that a wide therapeutic window exists. Finding a therapeutic window between cancerous cells and normal cells is crucial for development of novel therapies. Furthermore, these findings may be applicable to a wider variety of cancers, as neurofibromin loss is found in breast cancer; gastrointestinal stromal tumors; neuroblastomas; rhabdomyosarcoma; and juvenile myelomonocytic leukemia; and others [32].

Early intervention is crucial for successful treatment of cancer, and clinical management of pNF is no different. Plexiform neurofibroma develops from Schwann cells after biallelic loss of neurofibromin, which is considered one of the “first-hits” during oncogenesis [33]. Therefore, AgNPs are in the unique position to treat not only established pNF, but also to eradicate theoretical pNF “progenitor” cells that have undergone biallelic loss of *NF1*, but have not fully transformed. This is evidenced by our finding that simply reducing expression of neurofibromin is sufficient to sensitize both NF1-associated Schwann cells (Figure 5A), and Schwann cells that are wild-type for neurofibromin, to AgNP [18]. To the best of the author’s knowledge, there is no other therapy in preclinical development that has the potential to target these tumor progenitor cells prior to tumor establishment (Figure 7). Prevention of plexiform neurofibroma tumor establishment, which in turn would prevent malignant transformation, would be a major breakthrough and could ultimately alter the clinical management of NF1 patients.

## 5. Conclusions

In summary, we found that neurofibromin-deficient plexiform neurofibroma were sensitive to AgNP at doses which had minimal effect on patient-matched neurofibromin haplo-insufficient Schwann cells. AgNP-mediated cytotoxicity is directly related to functional neurofibromin expression, as restoration of functional neurofibromin expression in pNF decreased sensitivity to AgNP and, conversely, knockdown of neurofibromin expression in NF1-patient-derived Schwann cells increased sensitivity to AgNP. Importantly, AgNPs were able to selectively eradicate pNF cells from co-culture with patient-matched Schwann cells. Our investigation shows evidence that a therapeutic window may exist for the use of AgNP as a treatment for inoperable pNF which does not respond to selumetinib, and may be useful as a prophylactic to prevent pNF formation. Based on our findings, more investigations are warranted into preclinical development of AgNP.

## Data Availability

The datasets presented in this study are available upon request from the corresponding author.
